# Pacemaker-Mediated Tachycardia Triggered by Phrenic Nerve Stimulation During Cryoballoon Ablation of Atrial Fibrillation: A Case Report

**DOI:** 10.7759/cureus.94198

**Published:** 2025-10-09

**Authors:** Giorgi Papiashvili, Mirian Jintchvelashvili

**Affiliations:** 1 Electrophysiology, Jo Ann University Hospital, Tbilisi, GEO

**Keywords:** atrial fibrillation, cryoballoon ablation, pacemaker-mediated tachycardia, phrenic nerve stimulation, pmt

## Abstract

Pacemaker-mediated tachycardia (PMT) - also known as endless loop tachycardia - is a well-known phenomenon in dual-chamber pacemakers programmed in atrial tracking modes, such as DDD or VDD. The initiating triggers of PMT are also well recognized and most frequently include ventricular premature beats (VPBs) or loss of atrial capture.

In this report, we describe a case where an unusual trigger - such as a stimulus delivered in the superior vena cava (SVC) for testing phrenic nerve capture - initiated PMT.

## Introduction

Pacemaker-mediated tachycardia (PMT) is a known phenomenon in dual-chamber pacemaker patients and can cause significant symptoms, including palpitations, fatigue, or exacerbation of heart failure [1-3]. Modern pacemakers employ algorithms to avoid PMT and to terminate it if it still occurs [1,4]. There are specific programming recommendations that help decrease the likelihood of PMT occurrence [5]. The usual triggers of PMT are also well established, and most frequently include either premature ventricular beats or loss of atrial pacing capture [6].

Pulmonary vein (PV) isolation is a cornerstone of the catheter ablation procedure to treat paroxysmal atrial fibrillation [7]. Cryoablation using an expandable balloon is a widespread technique for PV isolation, which involves freezing the PV ostia [8]. Since cryoablation of the right-sided PVs carries a risk of right phrenic nerve damage due to its proximity to the right PVs, it is common practice to place a diagnostic catheter inside the superior vena cava (SVC) and pace at high output to capture the right phrenic nerve, monitoring diaphragmatic contractions during right-sided freeze cycles [8].

In this report, we present a case of PMT that was repeatedly initiated by an unusual trigger, such as electrical stimulation delivered in the SVC to test right phrenic nerve capture during cryoballoon isolation of the right-sided PVs.

## Case presentation

A 71-year-old male patient was admitted for scheduled PV isolation with cryoballoon. His past medical history included arterial hypertension, diabetes mellitus, chronic coronary artery disease with coronary angioplasty, and dual-chamber pacemaker (Sustain XL DR; Abbott Laboratories, Abbott Park, IL, USA) implantation for sick sinus syndrome and paroxysmal atrial fibrillation (tachy-brady syndrome). The patient’s medications included enalapril, metoprolol, atorvastatin, rivaroxaban, amlodipine, amiodarone, and metformin.

The patient presented for the procedure in a dual-chamber paced rhythm, with ventricular pacing artifacts coinciding with the onset of the conducted QRS complex and causing mild fusion. His echocardiography revealed normal left ventricular systolic function, mild left atrial dilation, and mild mitral regurgitation. The pacemaker was programmed in DDDR mode, with a base rate of 60 bpm, sensed atrioventricular (AV) delay of 225 ms, paced AV delay of 250 ms, and post-ventricular atrial refractory period (PVARP) of 300 ms.

The ablation procedure was performed under general anesthesia. Through the right femoral venous access, a steerable decapolar catheter was placed into the coronary sinus, and a fixed-curve quadripolar catheter was placed at the His bundle region. Transseptal puncture was performed under fluoroscopic and intracardiac pressure control, and the FlexCath Advance sheath (Medtronic CryoCath LP, Quebec, Canada) was placed into the left atrium. After isolating the left-sided PVs with the cryoballoon (Arctic Front Advance, Medtronic CryoCath LP, Quebec, Canada), the quadripolar catheter was moved to the SVC for phrenic nerve pacing. This is usually performed to monitor phrenic nerve capture during freezing of the right-sided PVs, which helps avoid right phrenic nerve damage. Upon starting stimulation from the SVC, a fully paced ventricular rhythm emerged with a rate of 90 bpm, and atrial activation occurred after each paced beat. The analysis of this rhythm was consistent with PMT. It always started immediately after the first stimulus delivered in the SVC, followed by the ventricular pacing. The interval from the SVC stimulus to the next ventricular pacing spike was exactly the programmed sensed AV interval. The ventriculo-atrial (VA) interval after the paced ventricle was outside the programmed PVARP. This reproducible pattern would have been incompatible with other mechanisms, such as atrial tachycardia.

**Figure 1:**
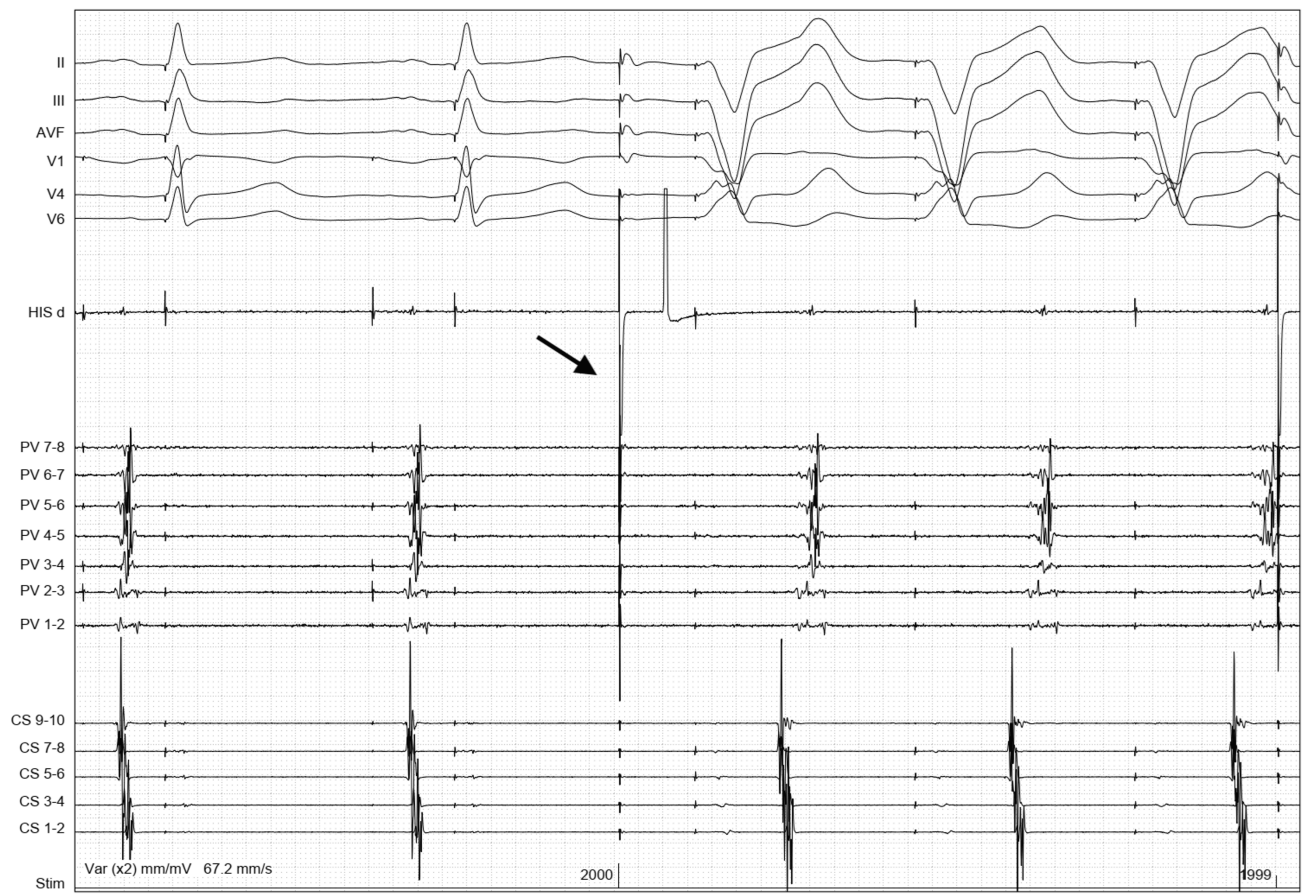
Initiation of pacemaker-mediated tachycardia following stimulus delivery in the SVC for phrenic nerve capture. The picture shows six ECG leads, together with some intracardiac EGMs. The catheter labeled HIS d is placed in the superior vena cava and is used to pace for right phrenic nerve capture. The channels labeled PV 1-2 to PV 7-8 are from the circular mapping catheter placed inside the right inferior PV, and they show the PV potentials before starting an application of cryo energy. CS 1-2 to CS 9-10 channels represent the EGMs from the decapolar catheter placed in the coronary sinus. On the left side of the picture, we see two atrial paced beats, followed by relatively narrow QRS complexes with the pacing spike at the beginning of each, causing mild fusion or pseudo-fusion. Then, we see a tall pacing spike (arrow), representing the stimulus artifact from the catheter placed in the superior vena cava. This spike is followed by a fully paced QRS complex after which there is an atrial activation, which can be appreciated both on the CS and PV electrogram channels; then again, the same paced QRS complex, and this pattern continues. ECG, Electrocardiogram; EGM, Electrogram; SVC, Superior Vena Cava; PV, Pulmonary Vein; CS, Coronary Sinus

The inspection and analysis of the tracings suggested that the pacing stimulus from the quadripolar catheter, delivered in the SVC, was picked up by the pacemaker and interpreted as a sensed atrial event (Figure 1). This triggered ventricular pacing after the programmed sensed AV interval, and subsequent VA conduction initiated PMT.

The procedure was completed without complications, and all four PVs were successfully isolated after temporarily reprogramming the pacemaker to a single-chamber pacing mode, as we decided that this was an easier and quicker temporary solution rather than letting the pacemaker's anti-PMT algorithms intervene.

## Discussion

PMT is a type of reentrant tachyarrhythmia that occurs in patients with dual-chamber pacemakers programmed in atrial tracking modes [1]. The prerequisite for PMT to occur is the presence of retrograde VA conduction. When the retrograde conduction is sufficiently slow to be picked up by the pacemaker’s atrial channel outside the programmed PVARP, it can trigger a subsequent ventricular pacing stimulus, which also gets conducted retrogradely, and the cycle repeats, thus initiating the type of tachycardia where the atrium is sensed and the ventricle is paced [1].

PMT often occurs at the programmed maximum tracking rate but can also occur at slower rates [9]. The retrograde conduction, which can be sensed by the atrial lead, is most often initiated by premature ventricular beats, because the retrograde conduction after ventricular premature beats (VPBs) is often decremental, or by the loss of atrial capture [6], which does not produce atrial depolarization; therefore, the subsequent ventricular paced beat with retrograde conduction is more likely to cause atrial depolarization. The rate of PMT depends on the retrograde conduction time, the programmed A-V interval, and the maximum tracking rate. 

PMT can also be triggered by atrial premature beats with prolongation of the AV delay to conform to the programmed upper rate interval being longer than the total atrial refractory period [6,10], or by sensing of a far-field signal by the atrial lead, usually the far-field R wave [6].

In our case, the pacing stimulus from the quadripolar catheter, delivered in the SVC, was sensed by the atrial lead, triggering the ventricular stimulus at the end of the programmed AV delay. The impulse from the ventricle was then conducted retrogradely via the conduction system, and since the atrium was not refractory due to the absence of atrial depolarization before the ventricular stimulus, this retrogradely conducted impulse was able to depolarize the atria and be sensed by the pacemaker's atrial lead, thus initiating the PMT. This is an interesting form of atrial oversensing that triggers PMT.

We think that physicians should be aware of such a possible scenario and be ready to intervene. In some cases, it may even be reasonable to consider temporarily reprogramming dual-chamber pacemakers to non-tracking mode before the cryoballoon ablation of atrial fibrillation.

## Conclusions

PMT is a known phenomenon in patients with dual-chamber pacing. Although the usual mechanisms of its initiation are well-known, some less common triggers can still be encountered in clinical practice, and clinicians should be able to recognize these scenarios. Hereby, we describe a case where an external electrical stimulus was erroneously interpreted by the pacemaker as a sensed atrial event, leading to the initiation of PMT.
